# Dr. W. Poyntell Johnston’s Clinic

**Published:** 1843-01-21

**Authors:** Henry Sargeant


### CLINIC OF DR. W. POYNTELL JOHNSTON.

*At the Locust Street Dispensary, Jan.* 14, 1843.

[Reported from notes by Henry Sargeant.]

### Lecture I.

Case I. — *Capsulo-lenticular cataract of the left eye—arborescent—very slightly tremulous, the result of a blow.* John Donahoe—æt. 20—has always enjoyed excellent health; has never been attacked with syphilis, scrofula, or any serious disease, other than those incidental to childhood, with the exception of occasional slight erysipelas of the face. This is always accompanied by considerable œdema, but promptly disappears without treatment. You perceive, gentlemen, that the face is at present very slightly puffed; this is the result of an attack which he experienced last week.

About eight years since, he received a blow upon the left eye, with a stick, which was followed by violent inflamation. Upon the subsidence of this, he discovered that he was entirely blind of the left eye. A partial vision was gradually restored; and at the end of a few months, he was enabled not only to distinguish the light, but even to recognise indistinctly the outline of large objects, particularly when these were presented to him laterally. From that period to the present, the disease has remained stationary.

Upon examining the eyes at a short distance, you perceive nothing anormal. They are of the same size—equally renitent. The conjunctiva offers no injection, and the cornea in each is perfectly transparent. The iris of the left eye, presents the same bluish gray color, and the same perfect polish as that of the opposite side. Upon a nearer examination however, you will perceive that the pupil is much more mobile in the left eye; that its motions are much more rapid and extensive than in the right, but that it is not equally regular. It is polygonal with very obtuse angles, and its margin presents at different points a fringed appearance of a black color.—From these fringes, two or three apparently delicate black threads may be seen running backwards to an opaque mass, which is situated behind the pupil.—These threads are not rendered completely tense when the pupil is dilated by the abstraction of light, but they appear flexuous when it is contracted. They do not interfere with the motions of the pupil, as these are more free than in the opposite eye. This opacity, which is separated from the iris by a certain interval, consists of three or four irregular patches of a clear or slightly clouded blueish white color; between these, and apparently more deeply seated, you will observe a more opaque matter, of an uniform cretaceous appearance. The whole mass thus constituted, appears broken into fissures by black lines, which run over its anterior surface, and give to it an arborescent appearance. The superior margin of the opaque body is more distant from the iris than the inferior; its plane offers, with that of the iris, a very slight angle. Sudden motions of the head appear to contribute a slight movement to the opaque mass. This appearance may, however, be illusory.

We have here, gentlemen, a *cataract*, as shown by the existence of an opaque mass in this situation: *a capsular cataract*, as exhibited by the more superficial blueish white patches; but not a *simple* capsular cataract; the more opaque mass, seen in the interval of these patches and behind them, indicate that the

*lens* itself is also affected. The *cataract*, therefore, is capsulo-lenticular.

But again—the black lines radiating over its surface, and giving the cararact an arborescent appearance, entitle it to another name: it is, to sum up all its characters, an *arborescent*, or *dendroid*, or *choroid capsulo-lenticular cataract.* These black lines are in the present instance, obviously due to the pigmentum of the uvea; they can be followed directly from the lines emanating from the black fringed pupillary margin of the iris. This species of discoloration of the opaque anterior capsule, has been ascribed to a prolongation of the choroid body over its surface, and also to such an enlargement of the red vessels in a capsulitis, as should render them distinct. In the present case, their origin is, we think, obvious; for the cellular adhesions by which they are connected to the iris, are also colored by the same pigmentum.

But again—the lens is situated at a greater distance from the iris than the lens of the opposite side; that is to say, the posterior chamber is larger than natural. To what are we to attribute this? We are aware that such should be the case in a *hard cataract*; because the hardness of a cataract depends upon the loss of its fluid particles, and the consequent closer approximation of those which are solid. Hence a *hard* cataract is diminished in size antero-posteriorly, as well as in all other directions, and proportionally retires from the iris; but the case before us offers no evidence of hardness or diminution of size, and we are compelled to look to some other cause for a phenomenon which really exists. The opacity was produced by a shock, not acting directly upon the occular globe, but upon the orbit. Hence we can understand how the lens might have been mechanically detached from its adhesion. Might it, under these circumstances, have been slightly driven backwards? This is a question to be resolved. That the lens may from a similar cause be dislocated and thrown through the pupil partially into the anterior chamber, is beyond controversy. We have seen a case of this kind at the clinic of Sichel, in which the lower margin protruded thus into the anterior chamber, and in which, by a simple incision of the cornea, the lens was at once extracted.

The real existence of a greater distance between the capsule and the iris in this case, is not only evident to the eye, but is shown also by the extreme mobility of the pupil. The alternate contraction and expansion of the iris occurs in a free space. This mobility, gentlemen, is also of great importance in determining another question, which must always present itself in the study of a cataract, particularly in reference to the propriety of an operation. This question is, whether the cataract is connected with amaurosis. In amaurosis, the pupil may be dilated, as in the torpid form; or it may be contracted, as in the irritative amaurosis, but it can never have the play which is here exhibited. The extreme mobility would consequently in the present case be sufficient to decide the point. But we have other and more direct evidence, that the cataract here is not complicated with amaurosis; the patient, you perceive, can dimly distinguish the outline of my hand, when I hold it near his eye, between him and the light; and when I present it laterally to his eye, in an obscure light, you remark that he detects the slight movement of my fingers. The cataract is not complicated with amaurosis, nor with any other visible affection of either eye.

Should the eye, therefore, be operated upon? Other points enter into the determination of this question. We are told that a cataract produced by external injury, should not generally be operated upon; because we may always look for inflammation as the result of the injury; because it is possible that the lens may recover its transparency, and because it is possible that a displaced lens may be absorbed; but these possibilities exist only in the case of recent traumatic cataract. Here the opacity has existed for nearly eight years, without variation, all danger of irritation, as connected with the cause of the lesion is clearly passed, as is also every hope of a recovery of natural transparency. The absorbing powers of the fluid with which the displaced lens is in contact, have been fairly tested, and found wanting. No objection to an operation can possibly exist on the score of the traumatic cause which produced the cataract.

We find, then, no local complication—no general diathesis—nothing in the general condition of the patient to counter-indicate an operation. The operation is therefore decided upon. But the present extremely unfavorable weather—the distance of the patient from his residence, and above all, the existence during the last week, of an erysipelas of the face, which has not yet subsided, are entirely opposed to the propriety or justice of its being performed to day, especially as nothing is lost by delay. The operation is therefore deferred till the earliest favorable opportunity, when we will examine also to which species of operation preference should be given in the present form of cataract.

In the interval, in order to combine as many elements of success as possible, attention will be paid to his general health; his bowels will be kept free, and an attempt will be made still further to elongate, and if possible to break up the loose cellular adhesions between the pupil and capsule. This may possibly be accomplished by a forced dilatation of the pupil, by means of belladonna.

CASE II.—*Purulent ophthalmia, succeeding labour—reduction of inflammation—chronic conjunctivitis for five years—reappearance in its purulent form—destruction of right eye—vascular keratitis of left eye.*—Mrs. ———, æt 63. You perceive, gentlemen, that the intellect of the patient is of so low a grade, that it is impossible to obtain any satisfactory history of her disease. We learn, however, in reply to questions, that she can recall no serious disease prior to the present one; and that previous to the present attack, she was not subject to affections of the eye. About eight years since, shortly after confinement, she was attacked with a violent ophthalmia, which caused a profuse discharge, and for some time prevented her from opening either eye. This inflamation was with difficulty moderated, and never entirely disappeared. She continued to suffer with sore eyes, and was constantly compelled to use collyria; her vision, however, was unimpaired. Five years since, while making an application to the eye, she was startled by the news that her husband had fallen into a creek; she instantly hastened to the spot, and was subjected to great exposure. On the following day, her eye again became violently inflamed, the pain for five days depriving her of sleep. On the 6th day, the right eye burst; the left gradually improved, to its present condition, and has thus continued ever since.

The right eye, you perceive, is perfectly collapsed; the left retains its natural form. On everting the lower lid, which is devoid of eyelashes, the palpebræ conjunctiva is found roughened throughout its whole extent, particularly in the cul de sac, at the external canthus, it adheres to the ocular conjunctiva by a great number of strong cellular bands. The ocular conjunctiva is of a pretty uniform rose color, although its vessels are distinct. These vessels do not terminate at the margin of the cornea, but are continued in a more attenuated form over its whole surface. They do not constitute a complete membrane, but are to be found over every part of the surface; and in their interstices the cornea is seen to present a dull, unpolished aspect. The whole conjunctiva is more dry than natural; in fact, there can scarcely be said to exist any secretion. There is no pain, other than a sense of friction; and vision, although exceedingly imperfect, still exists sufficiently to enable her to move about.

This case, owing to the imperfect details afforded by the patient, need not occupy us long. The only point to which we wish to direct your attention, is the vascular condition of the surface of the cornea—*the vascular keratitis*. This is caused generally, and has doubtless been caused in the present instance, by the mechanical irritation which must result from the incessant friction of the roughened palpebral conjunctiva over the cornea in the act of winking, and during the movements of the eye. Not unfrequently, a simple catarrhal ophthalmia, become chronic, is connected with a granular condition of the palpebral conjunctiva, capable of thus vascularising the cornea. In the more simple cases, the *keratitis* will frequently disappear, after the removal of the exciting cause, viz: the granulations of the palpebral conjunctiva. These may be removed by the frequent application of the sulphate of copper, in the solid form; for the removal of vegetations, the knife or caustic is requisite. In the present case, the age of the patient—the violence of the preceding inflammation—the duration of the disease—the entire change in the character of the conjunctiva, which is dry, and tends to assume the character of skin, almost preclude hope of benefit by remedial means. The instillation, night and morning, of a few drops of the aqueous solution of opium, will probably afford as much relief as any other local application. Should the vessels multiply, and form a complete membrane over the cornea—should the dryness of the conjunctiva continue at the same time to increase, the disease would then be a *Xeropthalmia*, of the commencing stage of which, the present case presents an example.

CASE III.—*Variolous Ophthalmia of right eye at 7 weeks of age.—Obliteration of pupil.—Formation of artificial pupil.—Partial vision.—Complete amaurosis of left eye, from wound three years since.—Exophthalmia following section of internal and external recti muscles.*—Barnard Clark, æt. 24, was attacked with small-pox when seven weeks old; during the attack the right eye became inflamed, and was followed by almost entire loss of vision on this side. Two years since an operation for artificial pupil was performed upon this eye. Since the operation, the sight is slightly improved. Vision in the left eye was perfect until Christmas, 1839; about this time the organ was injured by a splinter. The patient cannot say at what point the eye was struck, but the accident caused some hæmorrhage, and was followed by smart inflammation. The inflammation slowly subsided, and, from this period, vision in the left eye gradually diminished. This diminution of vision was not attended by any prominence of the eye; upon this point the patient is positive, however we may vary our questions. Two years since the internal rectus muscle was divided, vision still existing to a moderate degree. The division of this muscle was followed by an inability to turn the eye inwards; to remedy this, the external rectus was divided two months afterwards, and four months after this the same muscle was divided a second time. After the division of these muscles, the remnant of vision in this eye rapidly diminished, and in a short time was entirely lost.

At present, you perceive that the right iris offers scarcely a trace of the original pupil; at the point where the pupil should exist a firm adhesion is formed between the iris and the anterior capsule of the lens. At the point of adhesion, the capsule is entirely opaque; at the inferior portion of its circumference, the iris has been detached by an operation, and presents an artificial pupil, by means of which the patient possesses sufficient sight to guide himself without assistance.

The prominence of the left eye is so marked that it must be obvious to all. With this extreme prominence is associated a fixed, staring look, which not only increases the deformity, but gives an imbecile, or half-idiotic appearance to an individual who, as you may judge by his replies, is by no means wanting in intellect. This deformity, of which he is conscious, is to himself a source of great annoyance.

The conjunctiva of the eye is moderately injected, beneath this the sclerotica presents at several points a bluish tint, and opposite to these bluish patches it appears attenuated, the pupil is enlarged so much that but a trace of the iris is visible—the portion of the iris which remains, is directed backwards, and through the dilated pupil it appears as if we could see the bottom of the globe which offers a dull concave reflexion.

There are two points in the case worthy of note. There is an entire obliteration of vision in the left eye following an injury. What is the character of this amaurosis? The nature of the injury—a splinter which penetrated the coats of the eye, as shown by the hæmorrhage and inflammation; this injury not followed by an abrupt loss of sight, but by a steady and uniform decrease of vision up to total blindness; this decrease of vision accompanied by an alteration of the sclerotica and the appearance of bluish patches, and this latter coinciding with a dull reflexion from the bottom of the eye,—all seem to indicate that the amaurosis is dependent not upon a primary affection of the retina, either torpid or irritative, but rather upon a disease of the choroid membrane of the eye—upon a *choroiditis* which has become chronic, and has terminated, or is disposed to terminate in degeneration. This diagnosis will account for all the phenomena, for the expansion and separation of the fibres of sclerotica through which the bluish tint of the choroid may be recognized, for a corresponding pressure inwards upon the retina, causing its attenuation, and producing gradually a paralysis which has now become complete. While this same attenuation will account for the dull concave reflexion which you perceive at the bottom of the eye. The amaurosis may be regarded in fact as a *chronic choroiditis* or commencing *glaucoma*.

The second point worthy of remark is the singular prominence of the left eye since the section of the internal and external recti muscles, together with the fixedness of the organ and the character of imbecility which it impresses upon the countenance. The power of the *internal rectus* to retain the globe within the orbit is you perceive entirely lost; if the patient is told to look towards his nose, he cannot even bring the eye to a straight position; it remains obstinately abducted. The analogous power of the *external rectus* is materially impaired; he is told to look outwards but cannot completely abduct the eye.

The disposition of the two oblique muscles to draw the globe forward is consequently only antagonized by two straight muscles, instead of four, and the balance of power is lost; the eye is therefore protruded, and being incapable of following the motions of the opposite organ, the deformity is extreme.

Now as like causes produce like effects, it is not improbable that similar cases of deformity, the result of sections of both muscles of the eye, will be presented when sufficient time has elapsed to enable us to judge correctly of the results of an operation which may still be regarded as a recent one. For the prominence of the eye, and its want of harmony with the motions of the opposite organ, remedies would of course be of no avail. Can any thing be done to restore a certain degree of vision to the left eye? It is asserted by the London ophthalmologists that by the use of small doses of arsenic they have succeeded in permanently curing commencing cases of glaucona. We have seen the practice pursued according to their own formula, and faithfully persevered in, at the Clinic of M. Sichel, but in all cases the failure was absolute. How can we look for a restoration to functions of a retina, which is destroyed entirely by a degenerated choroid coat? Still as clinical instruction justifies the experimental use of remedies which can do no harm, even when we can anticipate no great benefit from their employment, and as the authority by which the assertion of cures effected under similar circumstances, is too high to be disregarded, the patient ought not to be deprived of what might by some be considered as a chance.

℞. Liquor. Potassæ Arsenitis, ℥ij.

S. Give five drops three times a-day, and gradually increase to ten drops.

☞We have been compelled, through want of space, to defer the remainder, comprising several cases, until our next.

